# Fungal endophytes boost salt tolerance and seed quality in quinoa ecotypes along a latitudinal gradient

**DOI:** 10.3389/fpls.2025.1602553

**Published:** 2025-06-09

**Authors:** Roberto Miño, Gabriel I. Ballesteros, Karina B. Ruiz, Ian S. Acuña-Rodríguez, Marco A. Molina-Montenegro

**Affiliations:** ^1^ Centro de Ecología Integrativa (CEI), Universidad de Talca, Talca, Chile; ^2^ Instituto de Ciencias Biológicas (ICB), Universidad de Talca, Talca, Chile; ^3^ Dirección de Investigación, Vicerrectoría Académica, Universidad de Talca, Talca, Chile; ^4^ Química y Farmacia, Facultad de Ciencias de la Salud, Universidad Arturo Prat, Iquique, Chile; ^5^ Centro de Investigación en Estudios Avanzados del Maule (CIEAM), Universidad Católica del Maule, Talca, Chile

**Keywords:** salt stress, protein content, halophyte, fungal endophytes, native microbiomes, latitudinal gradients, *Chenopodium quinoa*

## Abstract

Soil salinity threatens global food security, making salt tolerance a key agronomic trait. Quinoa (*Chenopodium quinoa* Willd.), a halophytic pseudo-cereal known for its high nutritional value, emerges as a promising candidate due to its inherent resilience to saline conditions. Although quinoa's physiological and morphological adaptations to salinity are documented, the role of native fungal endophytes in enhancing salinity tolerance remains largely unexplored, particularly across diverse genotypes. This study investigates the contributions of quinoa-associated endophytes to salinity tolerance and seed quality in different genotypes, thus contributing to understand ecological interactions bolstering crop resilience. To achieve this objective, five quinoa genotypes were selected based on their distribution along a 2,200 km latitudinal gradient (19°–39° S), representing a range of ecological niches. Plants with (E^+^) and without (E^−^) fungal endophytes were subjected to salinity treatments of 0, 200, and 400 mM NaCl. Salinity tolerance was assessed through photochemical efficiency, gene expression analysis of C*q*NHX1, and plant survival rates. Seed quality was evaluated by measuring seed weight and protein content, providing a comprehensive assessment of the endophytes' impact on quinoa under stress conditions. Our results reveal that native microbiomes significantly enhanced salinity tolerance and seed quality in a genotype-dependent manner. Notably, E^+^ plants demonstrated improved photochemical efficiency and higher expression levels of C*q*NHX1 under high salinity conditions, with survival rates increasing by up to 30% compared to E^−^ plants. Seed weight and protein content were also positively affected, with E^+^ plants showing up to a 25% increase in protein content under 400 mM NaCl stress. Remarkably, E^+^ plants exhibited no negative effects under non-saline conditions. These findings suggest that fungal endophytes interactions shift from neutral to beneficial under salinity, with no trade-offs under normal conditions. This highlights the potential role of endophytes in enhancing quinoa resilience and nutritional value, reinforcing their importance for crop adaptation in the face of climate change. Future research should explore the molecular mechanisms underlying these beneficial interactions and assess their applicability to other crops, paving the way for innovative strategies in plant breeding and conservation.

## 1 Introduction

Climatic conditions significantly influence agricultural productivity, affecting agroecosystems globally (Bilalis et al., 2019). Yet, these systems face increased challenges from environmental stressors, such as drought, high temperatures, and soil salinity, which are exacerbated by climate change (Okon, 2019; Bao et al., 2023). These are projected to become more severe globally forecasted to intensify in most parts of the world, reducing crop yields, survival, and quality, thus impairing food insecurity (Choukr-Allah et al., 2016; Abdel Latef et al., 2021; Ballesteros et al., 2023; Filho et al., 2023). In parallel, the growing global population further pressures agricultural systems to enhance crop yields and resilience to climate change (Weil et al., 2021). Identifying naturally drought and salt-resistant plant species, as alternative sources of edible products, such as leaves, sprouts, or grains, is vital to mitigate the impacts of climate change (Burritt, 2019; Hlásná Čepková et al., 2022; Pathan and Siddiqui, 2022).

Quinoa (*Chenopodium quinoa* Willd.), an Andean grain crop, emerges as a promising food source due to its gluten-free seeds, high protein content, and resistance to multiple abiotic stresses (high salinity, drought and frost) (Bazile et al., 2014; Li et al., 2021). Indeed, quinoa is well adapted to grow under marginal or extreme environmental conditions, where conventional crops falter (Zurita-Silva et al., 2014; Choukr-Allah et al., 2016; Hinojosa et al., 2018). It has a wide natural variability and genetic diversity, which stems from 7,000 years of domestication and adaptation to a wide range of edapho-climatic conditions and latitudes (from 11°N in Colombia to 40°S in Chile), with five different ecotypes and thousands of accessions (García-Parra et al., 2020). In Chile, there are two ecotypes of quinoa (*Salares/Salt flats*, and Coastal*/*Lowlands) cultivated in three isolated, fragmented production zones along a latitudinal gradient (19.7°S to 40°S) (Fuentes et al., 2009; Cai and Gao, 2020; Patiranage et al., 2022). This unique distribution of quinoa along a clinal gradient showcases its remarkable ability to adapt to diverse environmental conditions; it exhibits broad plasticity in terms of morphology, and can adapt to altitude, drought and salinity (Bazile et al., 2014; Martínez et al., 2015). Therefore, quinoa offers to be a promising crop to endure the increasing drought and salinity conditions under the global climatic change scenario (Cai and Gao, 2020).

Quinoa is considered as a facultative halophyte, thriving in saline soils. It has a high salinity tolerance, from 150 mM NaCl to up to 750 mM NaCl (Orsini et al., 2011; Adolf et al., 2013; Pitzschke, 2016; Hussin et al., 2023). In contrast, salt-sensitive crops such as barley, wheat, and corn experience significant yield reduction when salinity exceeds 40 mM NaCl, limiting their cultivation in saline environments (Hinojosa et al., 2018). Its salt resistance is a complex trait involving multiple genes and a diverse array of physiological, morphological, and biochemical mechanisms operating at various levels (Flowers and Colmer, 2015; Cai and Gao, 2020; Hussin et al., 2023). These mechanisms include Na^+^ exclusion and K^+^ retention in the leaf mesophyll, production and accumulation of osmolytes (such as soluble sugars, proline, and polyamines), and compartmentalization of Na^+^ in vacuoles through vacuolar NHX antiporter proteins and epidermal bladder cells (Ruiz et al., 2016; Cai and Gao, 2020; Moog et al., 2022). In general, salt stress tolerance shows significant ecotypic and genotypic variation, with genotypes from the *Salares* ecotype generally exhibiting higher tolerance compared to Coastal/Lowlands, which are more similar to glycophytic plants, like wheat (Bazile et al., 2014; Morales et al., 2017; Ruiz et al., 2019). Differences among genotypes are evident in cytosolic Na^+^ and K^+^/Na^+^ ratios, CO_2_ assimilation, germination rate, organic solute accumulation, and seedling growth under salinity stress (Ruiz-Carrasco et al., 2011; Ruiz et al., 2019; Cai and Gao, 2020). However, geographical distribution does not strictly determine salinity tolerance, as some lowland varieties may have similar or greater salt tolerance than *Salares* varieties (Schmöckel et al., 2017; Ruiz et al., 2017). In terms of nutritional-related parameters, quinoa seeds have a low content of sugar and calories; they present a high protein content with excellent balances of essential aminoacids, as well as high contents of fiber, lipids, carbohydrates, micro- and macronutrients (Granado-Rodríguez et al., 2021). Importantly, quinoa also exhibits a strong genotypic variability in seed quality-related parameters, including size, protein and mineral contents, which are also influenced by the environment (Miranda et al., 2011; Vega-Gálvez et al., 2018; Granado-Rodríguez et al., 2021).

Hence, the diversity observed among quinoa genotypes would constitute the primary factor enabling quinoa’s adaptability and growth across various ecosystems, while maintaining its productivity and nutritional properties, traits that would exclusively depend on its genomic information (Redman et al., 2022; Souri Laki et al., 2024). However, this perspective neglects the contribution of indigenous endophytic microbes to plants’ resilience and adaptation against environmental stress in complex habitats (Pitzschke, 2016; Redman et al., 2022). In quinoa, this topic has been largely underexplored, particularly in terms salt stress responses and seed nutrient content (González-Teuber et al., 2017, 2018, 2022). This is crucial, because if we wrongly attribute plant plasticity to either the environment or the genome, or the interaction of both, overlooking the influence of the associated microbial ecology, then our predictions about plants’ adaptation to future stresses are likely to be incorrect (Bolin, 2025).

Endophytes, which form symbiotic relationship with plants, are posited to significantly enhance quinoa’s adaptability to adverse conditions (Olivieri et al., 2021; Aizaz et al., 2023; Rétif et al., 2023). Through a complex array of mechanisms, endophytes may boost plant growth by improving nutrient uptake, nitrogen fixation and phytohormone production, thereby increasing yield and nutritional quality under stress. These microbial interactions would also increase plant yield parameters, even despite being challenged by stressful environments and/or harsh conditions (Aslam et al., 2020; Kaul et al., 2021; Osman et al., 2021; Omer et al., 2022; Badran et al., 2023). In quinoa, research on exogenic co-inoculation with specific fungal endophytes (*Talaromyces minioluteus* and *Penicillium murcianum*) has demonstrated beneficial effects in mitigating the impacts of drought and salinity. Yet, these studies were conducted on endophyte-free plants and limited to a unique genotype obtained from salt flats (González-Teuber et al., 2018, 2022). Hence, while the role of endophytic communities as enhancers of quinoa’s resilience to environmental stressors is an emerging area of interest, and some of the interactions have begun to be elucidated (González-Teuber et al., 2017), much remains to be explored. For instance, the diversity of indigenous, native endophytic communities across different quinoa genotypes, and their contributions in enhancing stress tolerance are largely unexplored, despite the growing body of research highlighting their beneficial influence on plant growth, yield and seed nutritional quality, particularly under abiotic stress conditions (Ballesteros et al., 2023). This knowledge gap is critical, given the realization that quinoa accessions from diverse eco-regions may host unique endophytes (U’Ren et al., 2024) potentially transmitted across generations, aiding for plants’ survival in harsh conditions (Molina-Montenegro et al., 2023). Concomitantly, macroclimatic variations, such as those observed across latitudinal gradients, are hypothesized to act as a driving force in shaping plant life-history traits. Consequently, it is proposed that such variations may also influence the incidence and structuring of fungal endophytic assemblages, affecting their diversity, abundance, and the nature of their symbiotic interactions with host plants (Arnold and Lutzoni, 2007; De Frenne et al., 2013; Mishcherikova et al., 2023; Molina-Montenegro et al., 2023). Yet, these interactions might not follow a strict latitudinal gradient, but be influenced by soil microbiota, plant species, host biomass and nutrient availability (De Frenne et al., 2013; Mishcherikova et al., 2023; U’Ren et al., 2024). Therefore, studying these dynamics across latitudinal gradients could clarify how indigenous endophytes help quinoa cope with abiotic stressors like drought and salinity.

The current study aims to investigate, under semi-controlled greenhouse conditions, the trends and variations in the symbiotic roles of endophytes among five quinoa genotypes from a latitudinal gradient (19°S-38°S), encompassing different ecosystems. The central hypothesis posits that the relative effects, conferred by native endophytes to quinoa, would be following a clinal trend, which would be reflected in shifts on their contributions, from a higher salinity stress tolerance to an improvement in seed nutritional quality and productivity. Specifically, we expected that the relative contribution of native endophytes, in terms of salinity stress tolerance, would have a negative clinal variation, being proportionally higher at lower latitudes, which would be reflected on differences in terms of physiological, biochemical, and molecular responses to salinity stress conditions. Conversely, we expected that endophytes’ contributions in terms of quinoa productivity and seed nutritional properties would exhibit a positive clinal variation, gradually increasing in quinoa from higher latitudes, compared to quinoa from lower latitudes. To test these hypotheses, quinoa plants naturally retaining their endophytes (control, E^+^) were compared with endophyte-free plants (treatment, E^-^), under different NaCl concentrations: control (0 mM NaCl), mild (200 mM NaCl) and high salinity stress (400 mM NaCl). To assess the differential effects of endophytes, the following traits were measured: plants’ survival rate, photosynthetic efficiency (F_v_/F_m_), seed weight, seed protein content, and the expression of *CqNHX1*, a gene encoding for a vacuolar Na^+^/H^+^ antiporter involved in Na^+^ sequestration. Based on the measured parameters, a relative endophyte contribution index model (RECI) was constructed, and used to estimate the relative contribution of endophytes on the tested genotypes under salinity stress conditions. Overall, this study not only aims to elucidate the potential of habitat-adapted symbiosis along clinal variations but also seeks to explore endophytes’ contributions across different quinoa genotypes, thereby enhancing our understanding of their ecological and agricultural significance.

## 2 Methodology

### 2.1 Removal of endophytes from quinoa seeds

Quinoa (*Chenopodium quinoa*) seeds from five genotypes, distributed along a 2,200 km latitudinal gradient were selected for this study: Pandela (19°S) and Paihuano (29°S) (both belonging to the *Salares* ecotype), and PRP (34°S), UdeC9 (35°S), and BO78 (39°S) (corresponding to the Lowlands ecotype). Prior to sowing, seeds underwent surface sterilization by immersion in 1% v/v sodium hypochlorite for 3 minutes, followed by three consecutive washes in sterile distilled water (1 min each). 100 seeds from each genotype were individually sown in 50 cm³ pots filled with a sterile 4:1 mixture of sand and peat. After two weeks of growth, seedlings were randomly assigned to two experimental groups: one retaining its native endophytic fungal community (E^+^), and the other undergoing an endophyte removal treatment (E^-^). To achieve this, all individual seedlings (E^+^ and E^-^) were first treated with a broad-spectrum systemic antibiotic (rifampicin, 50 µg mL^-1^) for one week to eliminate endophytic bacteria (Barrera et al., 2020; Hereme et al., 2020). Five days after the antibiotic treatment, a treatment to eliminate fungal endophytes was administered only for E^-^ seedlings, using the fungicide Benlate (2 g L^-1^; DuPont) at a concentration of 0.5 g L^-1^, for another week; the E^+^ group was treated with sterile water in equivalent volumes (Barrera et al., 2020; Hereme et al., 2020). After five days, and to verify the efficacy of the antibiotic and fungicide treatment, three seedlings from both groups were randomly selected, and five sections of their roots were cut using a razor blade. The effectiveness of the antibiotic treatment was assessed by checking for the absence of bacterial colony growth, through the culture of root samples on 10 petri dishes containing LB agar at 28°C for 5 days. In parallel, the antifungal treatment was verified using two different methods; half of the root samples assigned for antifungal evaluation were stained with trypan blue in an acid glycerol solution and inspected under microscope at 400x magnification (Motic BA410) observing only fungal hyphae in the E^+^ samples. The last set of root sections were incubated in potato dextrose agar plates (PDA, Difco) at 18°C for 2 weeks. Hence, after 6 weeks, E^-^ quinoa plants were deemed as fungi endophyte-free only when there was no outgrowth of fungi nor bacteria in the agar plates, thus being suitable for their usage in subsequent experiments.

### 2.2 Plants, growth conditions, and salinity treatment

Seedlings were maintained in a greenhouse, under natural light and temperature conditions (1324 μmol m^-2^s^-1^ ± 243, 20°C ± 4), and watered daily with 20 mL of sterilized water per pot. A total of 180 E^+^ and 180 E^-^ seedlings (N=360; 36 seedlings per genotype/endophyte condition) were transplanted into 10 L pots filled with a 4:1 mixture of sterile sand:peat and were randomly selected for salinity stress treatments, using the following concentrations: 0 mM NaCl (control), 200 mM NaCl, and 400 mM NaCl. The selection of these concentrations was based on the need to investigate the salinity response of *Chenopodium quinoa* across a range from non-saline to moderately and highly saline conditions relevant to its known tolerance (Jacobsen, 2003; Koyro and Eisa, 2008; Morales et al., 2011; Adolf et al., 2012; Ruiz et al., 2016). Salt stress treatment involved daily watering of E^+^ and E- plants with 200 mL of tap water. This was done using either plain tap water without NaCl (control, 0 mM), or tap water mixed with either 200 mM NaCl (for mild stress) or 400 mM NaCl (for severe stress). Therefore, for each genotype, the experiment included twelve replicates for each salinity level (0 mM, 200 mM, and 400 mM NaCl) and endophyte condition (E^+^ and E^-^) in a completely randomized block design. To minimize any potential block effect, all plants were rotated weekly.

### 2.3 Measurements of endophytes’ contributions to environmental tolerance traits

#### 2.3.1 Chlorophyll fluorescence (F_v_/F_m_)

To assess the effects of symbiotic status on plant photosynthetic performance of quinoa plants upon different salinity stress conditions, the maximum quantum yield of photosystem II was estimated by leaf chlorophyll fluorescence measurements. This parameter has been widely used to characterize the response to stress in different plant species (Hereme et al., 2020; Balboa et al., 2020). Briefly, using a pulse-amplitude modulated fluorometer (Pocket PEA, Hansatech Instruments Ltd, Norfolk, United Kingdom), the chlorophyll fluorescence ratio F_v_/F_m_ (where F_v_ refers to F_m_ – F_0_) was used to detect changes in PSII induced by salinity, on the tested genotypes with different symbiotic status (E^+^ and E). To ensure optimal photochemical efficiency, three fully expanded leaves per individual were dark-adapted for 30 minutes before being measured by means of a leaf clip. All measurements were conducted at noon.

#### 2.3.2 RNA isolation, cDNA synthesis and quantitative RT-PCR analysis of *CqNHX1* gene

Since NHX1 has been correlated with vacuolar compartmentation of Na^+^, we quantified the relative expression of *CqNHX1* using quantitative real-time PCR (qRT-PCR). Briefly, total RNA was extracted from shoots employing the PureLink^®^ Plant RNA Reagent (Invitrogen, USA) and remaining DNA was removed with the TURBO DNA-free™ Kit (Thermo Fisher Scientific, Waltham, MA, USA). Then, RNA integrity was assessed on agarose gels, and concentration measured with a NanoDrop One Microvolume UV-Vis Spectrophotometer (Thermo Fisher Scientific, Waltham, MA, USA). cDNA was synthesized using the First Strand cDNA Synthesis Kit (Thermo Fisher Scientific, Waltham, MA, USA). All kits were used according to the manufacturer’s protocols. Then, qRT-PCR was conducted using *CqNHX1* primers previously published for *C. quinoa* (5’-GCACTTCTGTTGCTGTGAGTTCCA-3’ sense; 5’-TGTGCCCTGACCTCGTAAACTGAT-3’ antisense) and using as reference the *Elongation Factor 1-a* houskeeping gene (*EF-1α*): 5’-GTACGCATGGGTGCTTGACAAACTC-3’(sense); 5’- ATCAGCCTGGGAGGTACCAGTAAT-3’ (antisense) to normalize and estimate up- or down-regulation of the target genes under salinity stress (Ruiz-Carrasco et al., 2011; Zhu et al., 2021). PCRs were carried on an Mx3000P thermocycler (Agilent Technologies. with Brilliant SYBR Green Master Mix (Stratagene, USA) following manufacturer instructions. Cycle threshold (Ct) values were obtained and analyzed with the 2^-ΔΔCt^ method (Livak and Schmittgen, 2001). Relative expression ratio (log_2_) and fold changes (FC) between *CqNXH1* and *EF-1α* were calculated from the qRT-PCR efficiencies and the crossing point deviation using the mathematical model of Pfaffl (2001) and is presented as the means (± S.D) of five biological replicates per genotype on each experimental condition including symbiotic status and salinity.

### 2.4 Measurements of endophytes’ contributions on performance traits

#### 2.4.1 Seed weight and protein content

In order to study the effects of symbiotic status on the improvement of quinoa genotypes’ performance upon different salinity stress conditions, the weight of one thousand seeds (SW in g) and the protein content were measured, as proxies of yield and nutritional seed quality, respectively (Granado-Rodríguez et al., 2021). Seed weight measurement was conducted using the standard procedure by FAO/ISTA (FAO & ISTA, 2023). First, the perigonium was removed by separating seeds from the chaff and straw. Then, four replicates were used, made up of 8 samples of 100 quinoa seeds with a diameter > 1.0mm. Seeds were weighed in a digital electronic scale (Boeco BBL-52; 0.01 g-precision) scale and multiplying the average by 10. These seeds were also used for estimation of soluble protein content (PC), using the method established by Bradford (1976) using bovine serum albumin (BSA, Sigma Aldrich) as a designated standard.

#### 2.4.2 Plant final survival

For each experimental group (endophyte presence/cultivar/salinity treatment; N=12), plant survival was recorded at the end of the experiment as a binary parameter, considering that the plant was dead if it presented more than 90% of damaged tissue (Baldelomar et al., 2019). Survival percentage was calculated as (S/N) x 100, where S is the number of plants that survived until the end of the experiment and N was the total number of evaluated plants per experimental condition (N=12).

### 2.5 Integrated estimation of the relative microbiome contribution on quinoa salinity stress tolerance and performance

Using tolerance and performance parameters (F_v_/F_m_, plant survival, seed biomass, protein content) an integrative equation representing Plants’ Responses (PR) was used, as a proxy to estimate the relative endophyte contribution index calculation (RECI) for all quinoa genotypes under salinity stress conditions (Montecinos et al., 2012; Balboa et al., 2020). The PR equation uses as input F_v_/F_m_, survival, and seed biomass parameters weighted by 0.3, as these were deemed as relevant parameters for crop production, while 0.1 was used as weight for protein seed content. As the PR equation used a scale of 0 to 1 to each parameter (Equation 1), the results of survival, biomass and protein content were divided by 100 or 10, respectively. Then, PR with and without endophytes (PR^E+^; PR^E-^) were used to calculate a Relative Endophyte Contribution Index [RECI; Equation 2].


(1) $$\text{PR}=\left[\left(0.3\times \;F_{v}/F_{m}\right)\;+\;\left(0.3\times \text{seed biomass}\right)\;+\;\left(0.3\times \text{Survival}\right)\;+\;\left(0.1\times \text{protein seed content}\right)\right]$$

(2) $$\text{RECI }\left(\%\right)=\left(PR^{E+}-PR^{E-}\right)\%$$

Where: PR^E+^ = Plant responses with symbiosis; PR^E-^ = Plant responses without symbiosis

Hence, RECI values closer to 0 indicate that the native microbiome had a lesser contribution in plants’ performance under different conditions, while positive values indicate a higher contribution of the native microbiomes on these parameters.

### 2.6 Statistical analysis

To evaluate whether the linear relationship between the latitude of origin of each cultivar and their responses to saline stress is influenced by the plant’s symbiotic status (E^+^ or E^-^), we fitted separate linear mixed models (LMMs) for each measured response variable: F_v_/F_m_, *CqNHX1* relative fold expression, seed weight, seed protein content, and final plant survival. In these models, symbiotic status (E^+^: with symbiosis; E^-^: without symbiosis), saline stress level (control, 200 mM, and 400 mM), and latitude of origin (19°, 29°, 34°, 35°, and 39° south) were included as fixed factors. The models were implemented using the “*lme*” function from the *nlme* R package (Pinheiro et al., 2024), with population treated as a random factor in the error structure. For each response variable, we compared the slopes of the fitted models between experimental groups using the “*emtrends*” function from the *emmeans* package (Lenth, 2025). In order to deeply explore the specific role of the symbiotic condition within each level of saline stress, new independent LMM models were also fitted only with the data that corresponded to a given condition (i.e., 0, 200 or 400 mM). To ensure the validity of the models, in all cases the normality of residuals was assessed using the Shapiro-Wilk test (R Core Team, 2024).

## 3 Results

### 3.1 Chlorophyll fluorescence F_v_/F_m_

**Figure 1:**
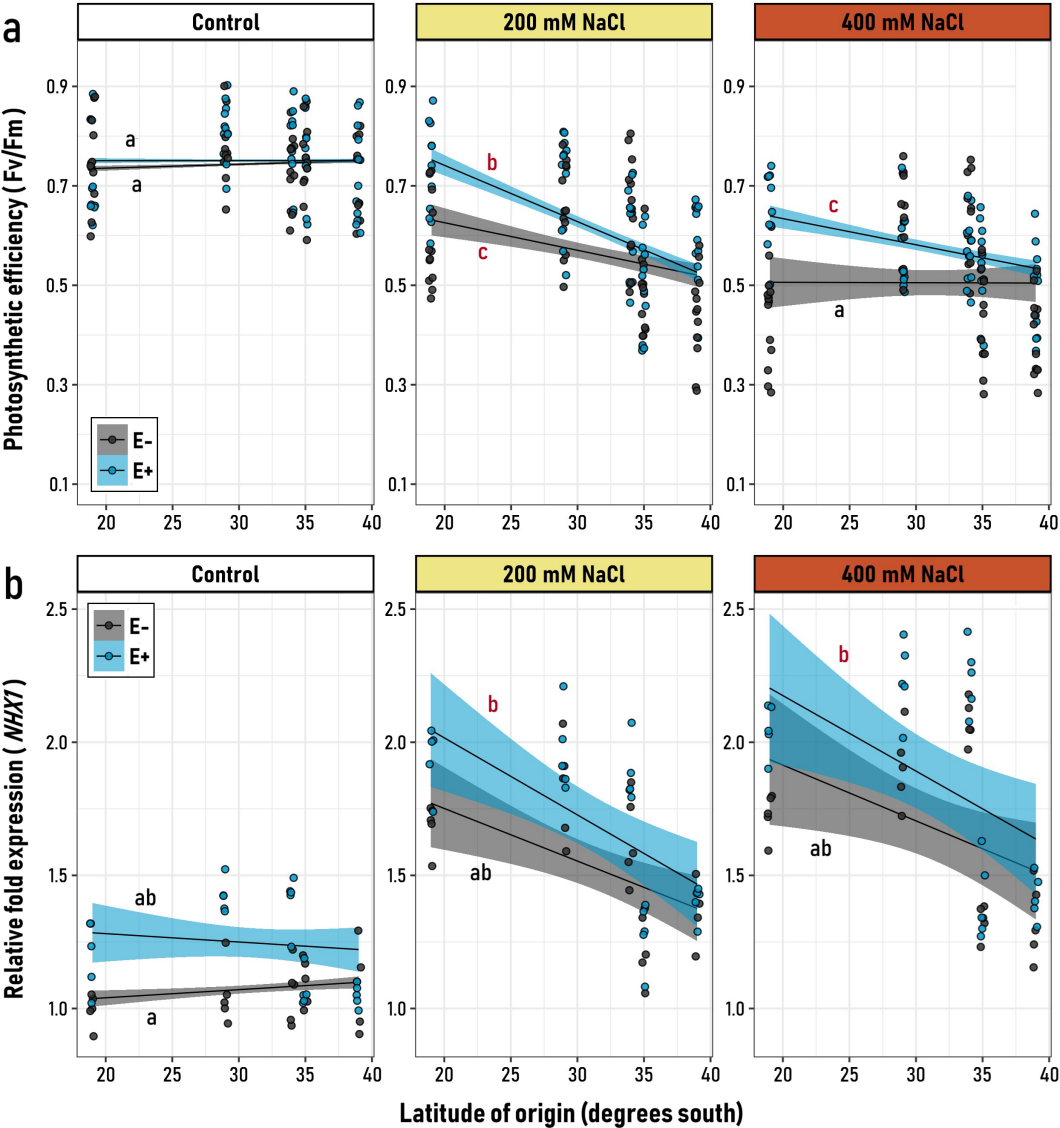
Relationship between the latitudinal origin of five *Chenopodium quinoa* genotypes (19° to 39° S) and **(a)** their photosynthetic efficiency (F_v_/F_m_), and **(b)** the relative expression of the *NHX1* gene, in symbiotic (E^+^, blue) and non-symbiotic (E−, grey) plants under varying levels of saline stress (Control, 200 and, 400 mM NaCl). Black regression lines represent the best-fit LMM models, with blue and grey shaded areas indicating 95% confidence intervals for E^+^ and E− groups, respectively. Letters on the plot denote model slopes: red for slopes significantly different from zero, and black for non-significant slopes. Pairwise comparisons of trends among the six experimental groups were conducted, with different letters indicating significant differences (*p*< 0.05, Tukey-adjusted).

In northern-origin plants, E^+^ individuals maintained a higher mean photochemical efficiency compared to E^-^ plants. This symbiotic advantage, however, diminished when comparing genotypes from southern latitudes, indicating that all experimental factors act as significant drivers of this response (**Figure 1a**, **Supplementary Table 1**). Under control conditions, the photosynthetic efficiency of the experimental plants, as described by F_v_/F_m_ monitoring, revealed that both E^+^ and E^-^ plants exhibited comparable photosynthetic performance (**Figure 1a**). This equivalence was consistent among genotypes from different latitudinal origins, as indicated by the non-significant slopes in both groups (black “a”). These results demonstrate that photochemical efficiency did not vary significantly with the latitudinal origin for either group in the absence of salt stress (**Supplementary Table 2**). However, at 200 mM NaCl, the influence of endophyte presence became evident, particularly among genotypes from northern latitudes. A similar pattern was observed under 400 mM NaCl, where E^+^ plants exhibited a negative and significant slope (red “c”), indicating differences in the capacity of genotypes from distinct latitudinal origins to tolerate saline stress (**Figure 1a**; **Supplementary Table 3**). In contrast, E^-^ plants showed no significant relationship with latitude (black “a”), suggesting that the impact of salt stress was uniform across all genotypes (**Figure 1a**). These findings underscore the adaptive role of microbial symbionts, particularly among quinoa genotypes originating from northern latitudes.

### 3.2 *CqNHX1* relative fold expression

As expected, both symbiotic (E^+^) and non-symbiotic (E^-^) plants displayed an overall increase in the expression of salt tolerance mechanisms, such as those regulated by the *CqNHX1* gene, when exposed to higher concentrations of NaCl (**Figure 1b**). Nevertheless, the non-significant triple interaction of the LMM model (Microbiome x Salt treatment x Latitudinal origin) suggests that the effect of the latitude was not detected in the overall general model (**Supplementary Table 1**). However, when plant responses were analyzed within each salt treatment, *CqNHX1* expression levels were more pronounced among plants from northern latitudes. This is evidenced by the negative, significant slopes observed in both E^+^ and E^-^ plants under 200 and 400 mM NaCl treatments (**Figure 1b**; **Supplementary Table 3**), which is similar to the trends observed in F_v_/F_m_ monitoring. Interestingly, the largest differences between symbiotic groups occurred under control conditions. Independent LMMs within each salt treatment revealed that E^+^ and E^-^ plants differed significantly in their mean responses only under control and 200 mM NaCl conditions (**Supplementary Table 2**). At 400 mM NaCl, the two groups were statistically indistinguishable (**Supplementary Table 2**). This suggests that, in the absence of stress, symbiotic quinoa plants (E^+^) exhibit a basal, constitutive state in which relative *NHX1* expression levels consistently surpass those of non-symbiotic plants (E^-^) across all latitudinal origins (**Supplementary Table 2**). However, as the level of stress increases, the relative expression advantage of E^+^ plants over E^-^ plants tends to diminish.

### 3.3 Seed weight and protein content

**Figure 2:**
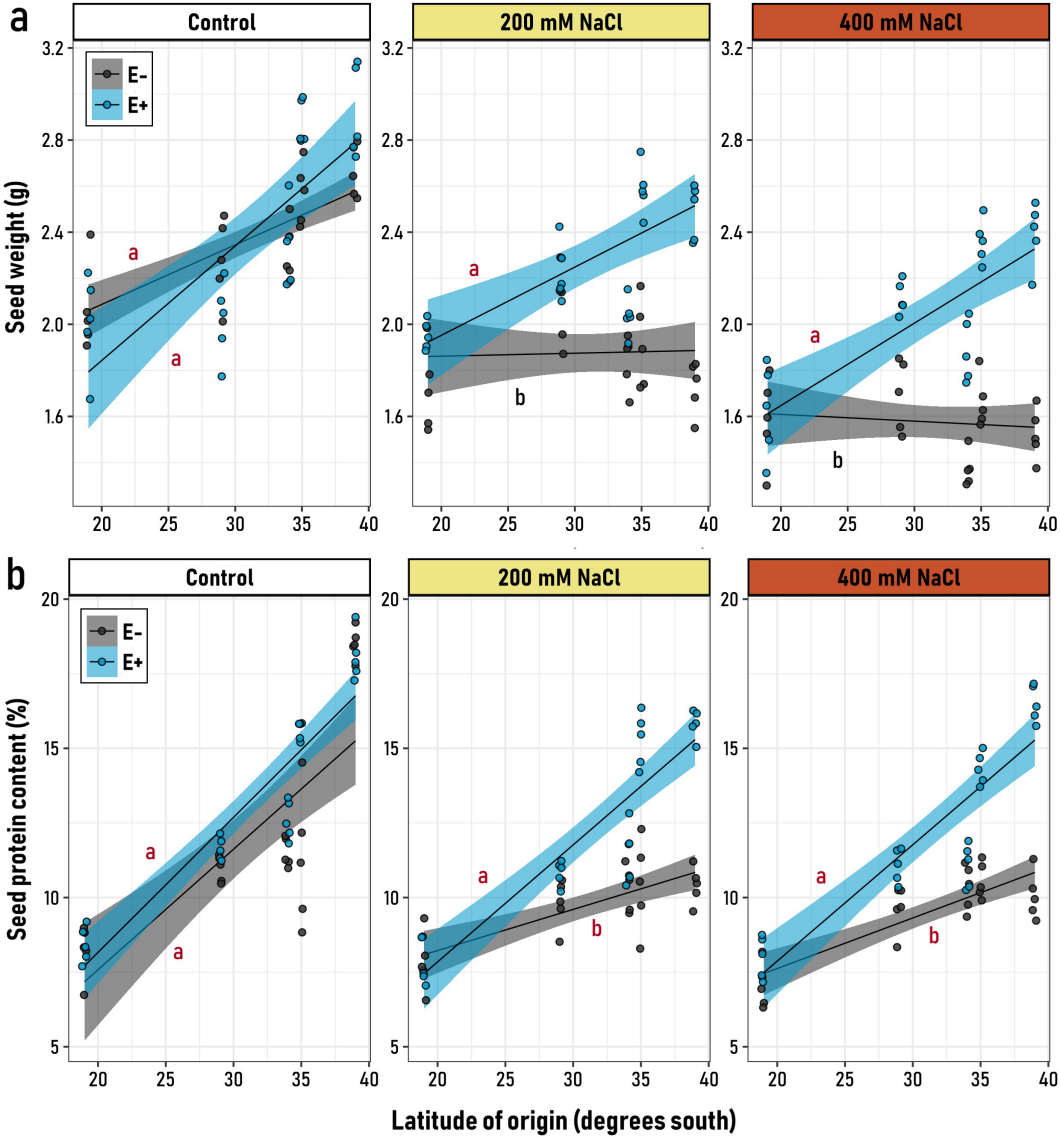
Relationship between the latitudinal origin of five *Chenopodium quinoa* genotypes (19° to 39° S) and **(a)** seed mean weight and **(b)** the seed protein content, in symbiotic (E^+^, blue) and non-symbiotic (E−, grey) plants under varying levels of saline stress (Control, 200 and, 400 mM NaCl). Black regression lines represent the best-fit LMM models, with blue and grey shaded areas indicating 95% confidence intervals for E^+^ and E− groups, respectively. Letters on the plot denote model slopes: red for slopes significantly different from zero, and black for non-significant slopes. Pairwise comparisons of trends among the six experimental groups were conducted, with different letters indicating significant differences (*p*< 0.05, Tukey-adjusted).

Both the microbiome and the salt treatment appeared as significant drivers of the seed weight and its protein content, however, contrary to seed weight, for the seed protein content the effect of these drivers was variable along the latitudinal gradient (**Supplementary Table 1**). Under control conditions, the latitudinal origin significantly influenced the seed weight of both symbiotic (E^+^) and non-symbiotic (E^-^) plants, with seeds from southern latitudes being ~30% heavier compared to the northern latitudes (**Figure 2a**). However, when plants were subjected to saline conditions, this pattern persisted only in E^+^ plants, as indicated by significant and similarly positive slopes (**Figure 2**). In contrast, E^-^ plants exhibited equivalent seed weights across latitudinal origins, characterized by non-significant (flat) slopes (**Figure 2**; **Supplementary Table 3**). However, as salt stress increased, the maintenance of this trait depended on the presence of symbiotic endophytes. Interestingly, quinoa seeds from genotypes at 19° S showed no clear effect of endophyte treatment on seed weight (**Figure 2a**), suggesting that in this cultivar, genetic factors might play a more significant role in determining seed weight.

Similar to the trends observed for seed weight, both E^+^ (with endophytes) and E^-^ (without endophytes) plants exhibited comparable positive slopes for seed protein content across the latitude of origin under control conditions (**Supplementary Table 2**). This indicates that, in the absence of stress, seed protein content was primarily determined by the cultivar rather than by the symbiotic status (**Figure 2b**). However, under saline conditions (200 and 400 mM NaCl), E^+^ plants maintained a strong, positive, and significant relationship between seed protein content and latitude. In contrast, E^-^ plants exhibited a weaker positive slope, which was significantly different from that of E^+^ plants.

### 3.4 Plant final survival

**Figure 3:**
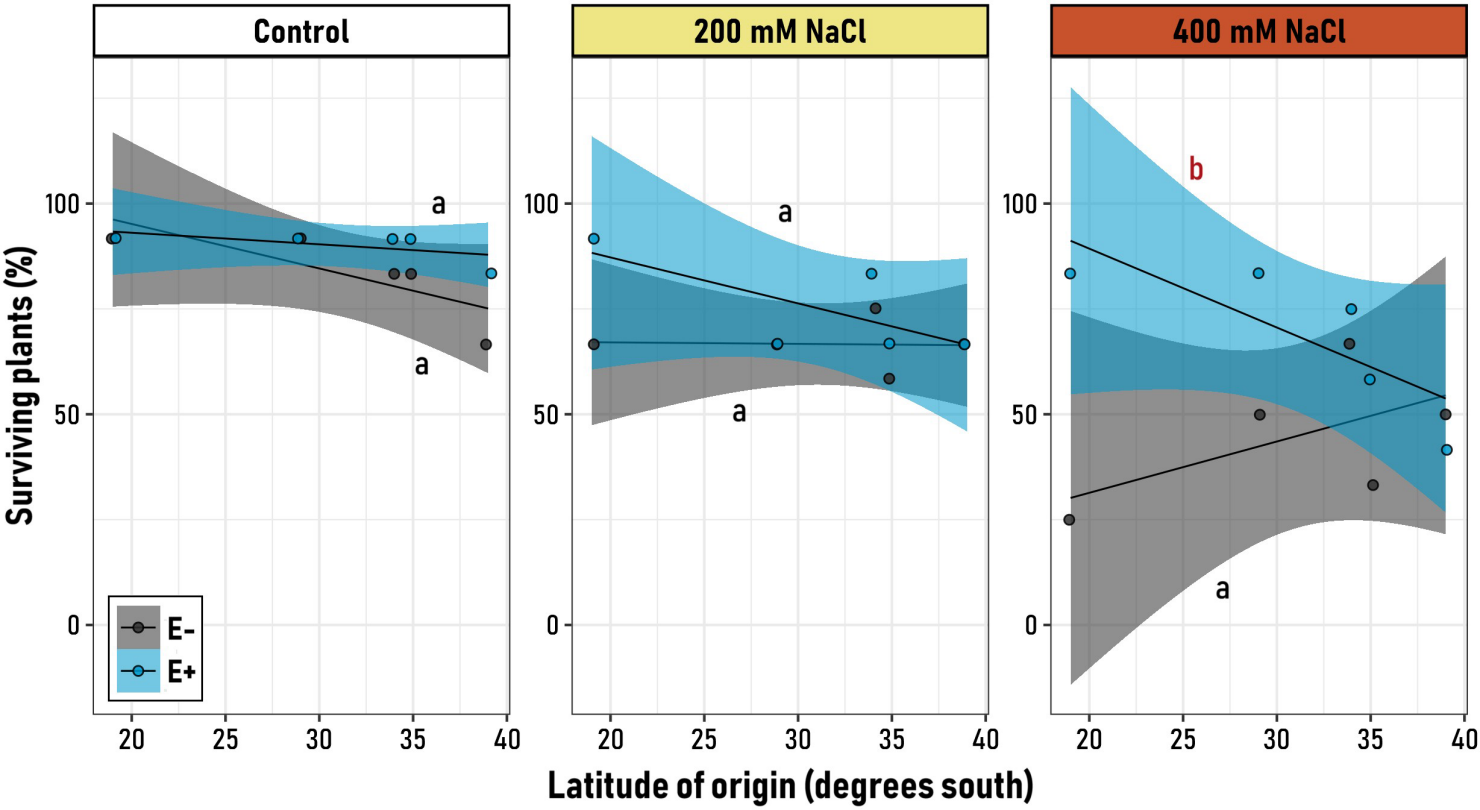
Relationship between the latitudinal origin of five *Chenopodium quinoa* genotypes (19° to 39° S) and the final proportion of survival plants, in symbiotic (E^+^, blue) and non-symbiotic (E−, grey) plants under varying levels of saline stress (Control, 200 mM NaCl, 400 mM NaCl). Black regression lines represent the best-fit LMM models, with blue and grey shaded areas indicating 95% confidence intervals for E^+^ and E− groups, respectively. Letters on the plot denote model slopes: red for slopes significantly different from zero, and black for non-significant slopes. Pairwise comparisons of trends among the six experimental groups were conducted, with different letters indicating significant differences (p< 0.05, Tukey-adjusted).

As denoted by the significant interaction of microbiome x salt treatment x latitudinal origin, the effect of the microbiome on the plant response to salinity stress in terms of survival was variable along the latitudinal gradient (**Supplementary Table 1**). Under control conditions, both E^+^ and E^-^ plants exhibited high survival rates, which were consistent across latitudes for both symbiotic groups, as evidenced by the non-significant (flat) slopes (**Figure 3**). Similarly, under moderate saline stress (200 mM), plants from both symbiotic treatments consistently showed high survival rates compared to control plants. However, it could be observed that the survival percentages of plants with (E^+^) and without (E^-^) microbial symbionts started to diverge at northern latitudes (19° S, **Figure 3**). This divergence became evident under severe saline stress (400 mM), where the higher differences among symbiotic and non-symbiotic plants can be observed in the northernmost originated cultivar (83.3% for E^+^ *vs* 25% for E^-^). Evidence of this is the significant negative slope of the symbiotic (E^+^) plant group at 400 mM of NaCl (**Supplementary Tables 2, 3**). Notably, the impact of microbial symbionts on survival varies among genotypes from different latitudinal origins, while becoming significantly different under highly stressful saline conditions (400 mM). Under these conditions, endophytes appear to be inconsequential for southern varieties, whereas they confer a survival advantage (and beneficial effect) to northern genotypes (**Figure 3**).

### 3.5 Integrated estimation of the microbiome contribution to quinoa performance upon salinity stress

**Figure 4:**
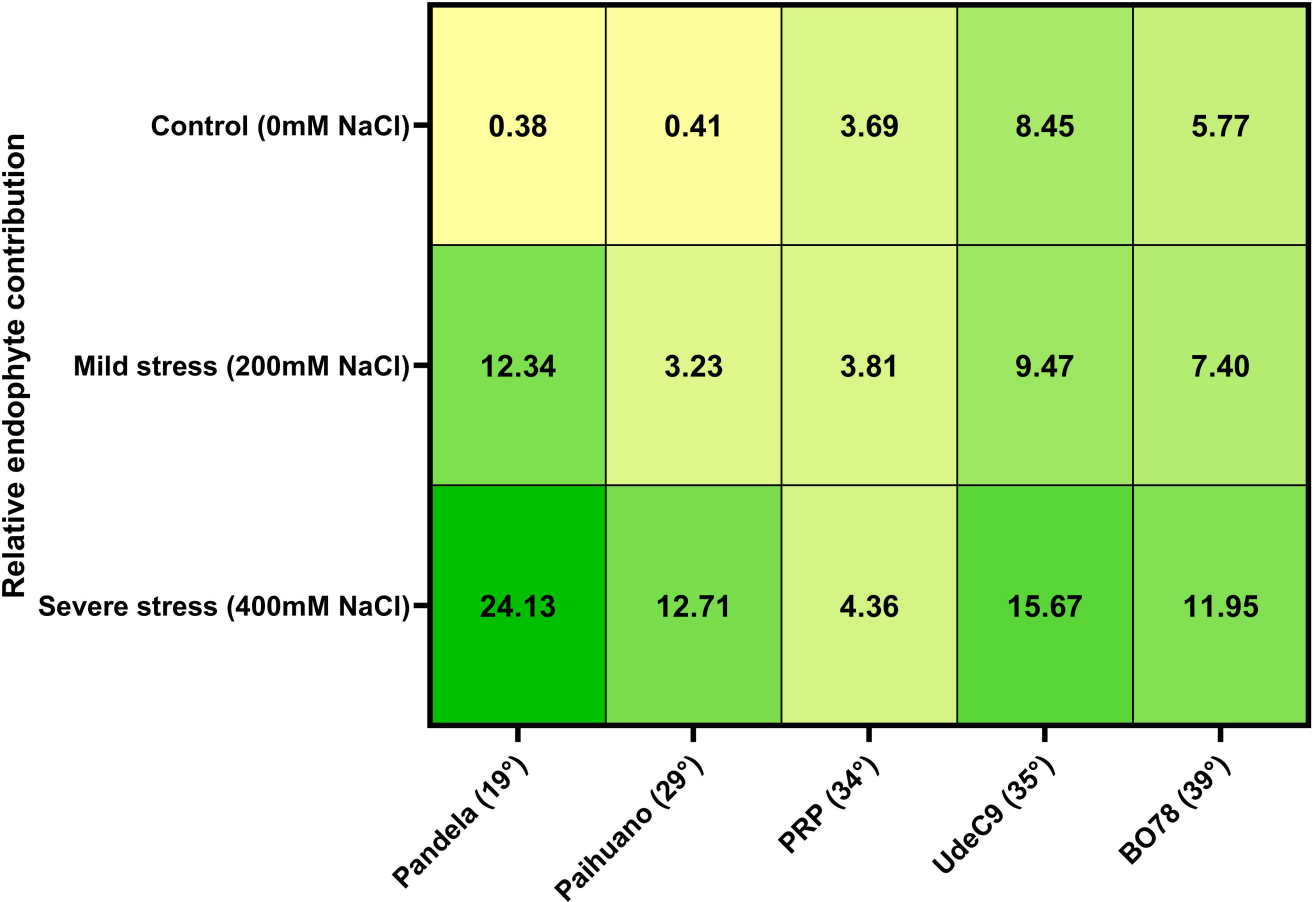
Relative endophyte contribution index (%) calculated using the RECI proposed model based on performance index (Equation 2). Higher numbers indicate a higher endophyte contribution.

Through the calculation of the Plant Response model (PR), a Relative Endophyte Contribution Index (RECI) was calculated for symbiotic (E^+^) and non-symbiotic (E^-^) plants under the three different salinity conditions (**Figure 4**; **Supplementary Figure 1**). According to this model, the presence of the native symbionts had a positive effect, albeit with different magnitudes, on all genotypes upon 200 mM and 400 mM NaCl. On the other hand, their contributions under control conditions would follow a positive relation with the clinal gradient. This suggests that the microbiome would play an important role in these parameters under salinity stress. When comparing genotypes, our results show that endophytes’ contribution would be similar for all treatments in PRP, but with a lesser contribution at 400 mM NaCl (4.36%), while at control condition, the observed endophytes’ contribution was similar to salinity stress conditions (3.69%). Therefore, PRP (coastal/lowlands ecotype) would be an average-performing genotype in control condition but would also be the best performing cultivar under the experimental salinity stress conditions used in this study. On the other hand, at 400 mM NaCl, Pandela (*Salares* ecotype) would be the genotype with the highest relative endophyte contribution (24.13%).

## 4 Discussion

Quinoa is a crop that can grow and survive under extreme environmental conditions (Jacobsen, 2003; Ruiz et al., 2015). While it has been used as a model plant to understand salt tolerance mechanisms (Ruiz et al., 2015; Manaa et al., 2019), to our knowledge, no prior studies have examined the beneficial presence of its native microbiome on relevant agronomic traits. Our results provide novel evidence suggesting a relative, differential endophyte contribution on quinoa plants, as variations in the endophyte-mediated effects on salinity stress tolerance, seed yield, nutritional content, and plant survival were observed along the latitudinal gradient. Considering that this study assessed both symbiotic status and salinity stress conditions in five genotypes, a novel index was used to estimate the relative endophyte contribution for each genotype and salinity condition; similar approaches have been used in previous studies to account for such differences (Montecinos et al., 2012; Balboa et al., 2020).

Remarkably, a positive, higher contribution of microbiome in salinity stress tolerance and plant survival was observed in genotypes from the northern part of the gradient (19S°-29°S; **Figure 3**), while in the southern part, a higher, significant role in terms of quinoa grain quality and seed productivity was observed (**Figure 2**). One possible explanation for these differential endophytes’ activity could be that genotypes from two different ecotypes were compared in this study (Salt flats/*Salares*, and Coastal/Lowlands). These are naturally cultivated in three isolated, fragmented production zones, including the extreme arid Andean northern highlands (ca 19.7°S), the saline and semiarid soils along the central coast (ca 34.3°), to the rainy-temperate southern regions (ca. 39°S, up to ca. 43°S). Along these zones, a highly contrasting transition of edaphoclimatic conditions is displayed, such as decreasing soil salinity and increasing rainfall (Bazile et al., 2014; Ayala et al., 2020; Ruiz-Carrasco et al., 2011; Miranda et al., 2012). Therefore, it is likely that the relative endophytes’ contribution to environmental tolerance, seed quality and productivity under saline conditions would be following an “*habitat-adapted symbiosis*” pattern (*sensu* Rodriguez et al., 2008). Presumably, the differential responses observed for all tested quinoa ecotypes would arise from endophytes’ previous exposure, adaptation and coevolutionary dynamics to selective pressures of the different ecosystems on which quinoa is found (Garnica-Díaz et al., 2023; Ballesteros et al., 2023; Bolin, 2025). These results are supported by differential levels of salt tolerance observed for endophytes isolated from halophyte plants, that could be due to their genetic background, or their interplay with their hosts (Manjunatha et al., 2022). It is plausible that quinoa responses would be mediated by their native endophytic communities rather than solely induced by the stressful environment. The latter uncovers a cryptic yet crucial role of native symbiotic endophytes in plant phenotypic responses to environmental fluctuations (Bolin, 2025).

Photosynthesis is among the most severely affected processes during NaCl salt stress exposure in plants, due to the accumulation of high levels of toxic Na^+^ and osmotically induced stomatal closure (Shabala et al., 2012; Manaa et al., 2019). One way to study the negative effect of NaCl in plants is by measuring the decrease of maximum quantum yield of fluorescence (F_v_/F_m_). In the case of facultative halophyte plants, such as *C. quinoa*, previous reports have shown non-apparent negative effects, or a slight, albeit significant reduction on leaf photochemical performance (F_v_/F_m_) for several genotypes upon salinity stress (from 100 mM to 400 mM NaCl), while also showing positive growth rates (Hariadi et al., 2011; Shabala et al., 2012; Manaa et al., 2019). Surprisingly, this study provides contrasting evidence in terms of F_v_/F_m_, as significant differences were observed for genotypes upon mild (200 mM NaCl) and severe salinity stress (400 mM NaCl), and that removal of endophytes is linked to a significant reduction in F_v_/F_m_ values, while also revealing a pattern aligned with the latitudinal gradient, upon mild and severe salinity stress, but not in control conditions (**Figure 1a**). These negative effects are likely explained by a decline of leaf pigments contents or instability in the pigment-protein complex (Badawy et al., 2021). Moreover, our results imply that endophytes would differentially mitigate the negative effects of saline stress on photosynthetic pigments, photosynthetic performance and PSII functionality, as these effects would be higher in genotypes from the northern part of the gradient (19° - 29°S) compared to genotypes from the southern part of the gradient.

Remarkably, in quinoa genotypes from the northern part of the gradient, symbiotic endophytes significantly induced the expression levels for the gene encoding for the proton-coupled Na^+^/H^+^ antiporter 1 (*CqNHX1*) compared to plants from the southern end, even under conditions of mild and severe salinity stress. CqNHX1 sequesters cytoplasmic Na^+^ into the vacuole or endosome, in exchange for H^+^ efflux to the cytosol, thus contributing to reduce Na^+^ toxicity, maintaining pH homeostasis in plant cells, and contributing to K^+^ uptake (Ruiz-Carrasco et al., 2011; Bassil and Blumwald, 2014). In quinoa, it has been shown that moderate salt stress (300 mM NaCl) elicits differential expression of *CqNHX1*, being higher on salt tolerant accessions, although transcriptional responses exhibit genotype-specific variations (Ruiz-Carrasco et al., 2011; Liu et al., 2024). Our study further elucidates the influence of native endophytes upon *CqNHX1* expression, revealing a pattern aligned with the latitudinal gradient. Specifically, in the case of the *Salares* ecotype, endophytes enhanced the expression levels of *CqNHX1* compared to their endophyte-free (E^-^) counterparts (**Figure 1b**). Conversely, in Coastal*/*Lowland genotypes subjected at severe salt stress conditions (400 mM NaCl), no significant differences in *CqNHX1* expression were observed between E^+^ and E^-^ plants (**Figure 1b**). Concomitantly, exogen inoculation using endophytes isolated from plants growing in saline environments showed an enhancement of *NHX1* expression and conferred salinity stress tolerance across various salt-sensitive crops, including pepper, tomato, lettuce, and onions (Acuña-Rodríguez et al., 2019; Molina-Montenegro et al., 2020; Ballesteros et al., 2023). Taken together, these results suggest a correlation between the presence of symbiotic endophytes, the higher expression of *CqNHX1*, and F_v_/F_m_ values. From an overall perspective, the observed differential contribution of endophytes in the enhancement of salinity stress tolerance are likely a result of co-evolutionary adaptations between endophytes and host plants, to cope with either dry or saline environments (González-Teuber et al., 2022).

Quinoa seed yield and nutritional quality are deemed as variable parameters, resulting from the interaction of genetic and multiple environmental factors (temperature, climatic condition, water status, soil nutrient content) (Chouhan et al., 2021; Granado-Rodríguez et al., 2021). While it has reported a relationship between agroecological conditions and environmental factors on nutritional traits of quinoa seeds (Granado-Rodríguez et al., 2021), there is still limited knowledge about the interaction between nutritional traits and symbiotic *status*. Despite this, it has been proposed that the associated microbiota would enhance plant growth under abiotic stress conditions, through different pathways, including higher production of primary and secondary metabolites, improved photosynthesis, better nutrient absorption, increases in antioxidant content, and greater antioxidant enzymatic activity (González-Teuber et al., 2022; Munir et al., 2022; Singh et al., 2023). Therefore, the negative effects derived from salinity stress would be counteracted by the presence of the native microbiome (Singh et al., 2023). In the case of severe salinity stress, Coastal*/*Lowland genotypes retaining their endophytes had a higher seed weight and protein content, compared to the endophyte-free condition, even despite having a reduction of ~18% in terms of photosynthetic performance (**Supplementary Figure 1**). On the other hand, the native endophytes from the *Salares* genotypes would not display a contribution in terms of quinoa productivity and nutritional quality. These results indicate that the role of endophytes in the symbiotic relationship turns from neutral to beneficial along the latitudinal gradient upon a higher level of salinity stress; this is reflected in the positive relation between seed weight and latitudinal gradient at 400 mM NaCl, suggesting that the native fungal endophytes would be contributing in a reduction of the metabolic costs involved in seed development (Carvalhais et al., 2013). One possible explanation to these observations is that genetic factors and morphological/physiological effects may cause significant differences in the endophytic communities during plant growth and development, particularly under high salinity conditions, thus leading to differential, beneficial effects (Wu et al., 2024). Remarkably, this plant-microbe interaction would not have a detrimental effect on seed weight and protein content, as similar values were observed for all genotypes under control conditions, indicating a neutral interaction (Hereme et al., 2020). Similarly, in wheat, inoculation of halophyte-isolated endophytes had a positive effect on these parameters compared to non-inoculated plants, while non-detrimental effects were observed (Manjunatha et al., 2022). Therefore, while the specific molecular mechanisms involved in these interactions remain to be elucidated, our results clearly point towards an increasingly positive contribution of endophytes under increasing salinity conditions along this latitudinal gradient.

Notably, while retaining endophytes provides differential advantages, these advantages would depend on the tested genotypes and/or their latitudinal origin. In the case of quinoa, this contribution would be higher in terms of salinity stress tolerance in the northern part of the gradient, and in terms of seed productivity from the southern part of the gradient (**Figure 4**). Hence, it is plausible that endophytes would be playing an important role in ecotypic differentiation, which is considered as one of the main strategies used by organisms to colonize and establish themselves in various environments (Molina-Montenegro et al., 2023). While geographic and environmental factors are known to favor ecotypic differentiation, microbes associated with plants may also play an important role in environmental responses (Liu et al., 2021; Bolin, 2025). This adaptive flexibility, which may promote plant-microorganism fitness alignments, not only enhances a plant’s capacity to navigate and thrive in various ecological niches but also underscores the pivotal role of the microbiome and its composition in the adaptation process. This interplay between genetic and molecular mechanisms of plants and microbiome composition is crucial, as it allows for a dynamic response to environmental challenges, thereby greatly influencing and elucidating the adaptation of certain ecotypes to their specific habitats (Fang et al., 2022; Ramandi et al., 2022). A recent study by Pang and colleagues (2020) further supports this perspective, revealing that both ecotype and environmental conditions can specifically restructure the microbiome, enriching certain hosts’ endophyte microbial taxa. This enrichment facilitates the microbiome’s significant contribution to the adaptation of various ecotypes to their respective environmental conditions. This is consistent with other studies, comparing microbial communities of ecotypes well-adapted to contrasting environmental conditions (Bowsher et al., 2020; Liu et al., 2021). Our study’s outcomes align with studies like those by Pang et al. (2020), suggesting that microbiome composition is integral to the adaptation of plant ecotypes to varying environmental conditions. Overall, and using the relative endophyte contribution index (RECI), a novel integrative model proposed in this study, we found an overall positive relationship between quinoa and its native endophytes. However, their contributions were distinctly influenced by their original habitat, which could be a consequence of differences in their putative, underlying associated molecular mechanisms and functional pathways encoded within their genomes (Partida-Martínez and Heil, 2011). This is especially true in the case of symbiotic endophytic relationships with quinoa plants from environments with higher salinity levels, such as the case of Pandela, on which endophytes contributions were higher compared to the other genotypes. On the other hand, and using the RECI model, we found slight, positive beneficial effects of endophytes for the PRP genotype, even under high salinity stress conditions. Moreover, and considering the plant response model, PRP would have a higher performance compared to the other genotypes, even without its endophytes (**Supplementary Figure 1**). This suggests that, in this genotype, plant-microbe interactions would be slightly positive or even neutral, as no negative effects were observed in the measured parameters.

## 5 Final remarks

Understanding and leveraging the role of plant-associated microbiomes is pivotal in enhancing crop productivity under environmental stress, a critical component in addressing global food security challenges. Quinoa, recognized as a superfood due to its exceptional nutritional quality and high stress tolerance, stands at the forefront of these efforts. Our study is pioneering in demonstrating differential contributions of native endophytes, from different genotypes across a latitudinal gradient, in terms of salinity tolerance and seed quality. These observations offer the possibility to conduct research on characterizing these native microbiomes in terms of their abundance, diversity and functional mechanisms, and to determine which would be key endophytes conferring these beneficial effects on both ecotypes. Moreover, these endophytes could also be isolated from these ecotypes, combined in novel bio-formulations or synthetic communities, and inoculated into quinoa and other crops, to further enhance their nutritional quality and tolerance to environmental stressors. This is a promising approach, particularly in the case of climate change scenarios, where productive ecosystems will become more arid and saline in future decades.

## Data Availability

The raw data supporting the conclusions of this article will be made available by the authors, without undue reservation.
