# Supplementary material for: Fungal endophytes boost salt tolerance and seed quality in quinoa ecotypes along a latitudinal gradient
Source: Front Plant Sci. 2025 Jun 9;16:1602553. doi: 10.3389/fpls.2025.1602553 (PMC12183269; doi:10.3389/fpls.2025.1602553)

SUPPLEMENTARY TABLE 1 | Analysis of variance (ANOVA) on the general LMM fitted models examining the relationship between each of the measured response variables (F_v_/F_m_, *NHX1* relative expression, seed weight, seed protein content and plant final survival) with the three main studied factors: the symbiotic status of the experimental plants (E^+^: with symbionts, E^-^: without symbionts), the level of experienced saline stress (Control, 200 and 400 mM), and the latitudinal origin (19°, 29°, 34°, 35°, and 39° S) of the five studied genotypes of *Chenopodium quinoa*. *d.f*.: degrees of freedom, *F*: F-statistic, *p*: probability value. A p-value less than 0.05 indicates that the observed effect of a factor or its interaction is statistically significant.

| Response | Factor | d.f. | F-value | p-value |
| --- | --- | --- | --- | --- |
| F_v_/F_m_ | (Intercept) | 1, 138 | 47899.79 | < 0.0001 |
|  | Microbiome | 1, 138 | 58.35 | < 0.0001 |
|  | Salinity | 2, 138 | 471.57 | < 0.0001 |
|  | Latitudinal origin | 1, 138 | 79.35 | < 0.0001 |
|  | Microbiome:Salinity | 2, 138 | 10.67 | < 0.0001 |
|  | Microbiome x Latitudinal origin | 1, 138 | 23.36 | < 0.0001 |
|  | Salinity x Latitudinal origin | 2, 138 | 38.93 | < 0.0001 |
|  | Microbiome x Salinity x Latitudinal origin | 2, 138 | 3.55 | 0.0298 |
| *CqNHX1* | (Intercept) | 1, 138 | 6502.639 | < 0.0001 |
|  | Microbiome | 1, 138 | 20.676 | < 0.0001 |
|  | Salinity | 2, 138 | 94.381 | < 0.0001 |
|  | Latitudinal origin | 1, 138 | 35.953 | < 0.0001 |
|  | Microbiome:Salinity | 2, 138 | 0.015 | 0.9856 |
|  | Microbiome x Latitudinal origin | 1, 138 | 1.94 | 0.1660 |
|  | Salinity x Latitudinal origin | 2, 138 | 8.937 | 0.0002 |
|  | Microbiome x Salinity x Latitudinal origin | 2, 138 | 0.026 | 0.9748 |
| Seed weight | (Intercept) | 1, 138 | 14959.021 | < 0.0001 |
|  | Microbiome | 1, 138 | 77.493 | < 0.0001 |
|  | Salinity | 2, 138 | 94.534 | < 0.0001 |
|  | Latitudinal origin | 1, 138 | 87.217 | < 0.0001 |
|  | Microbiome:Salinity | 2, 138 | 16.701 | < 0.0001 |
|  | Microbiome x Latitudinal origin | 1, 138 | 37.145 | < 0.0001 |
|  | Salinity x Latitudinal origin | 2, 138 | 8.602 | 0.0003 |
|  | Microbiome x Salinity x Latitudinal origin | 2, 138 | 0.78 | 0.4604 |
| Seed protein | (Intercept) | 1, 138 | 9318.395 | < 0.0001 |
|  | Microbiome | 1, 138 | 77.762 | < 0.0001 |
|  | Salinity | 2, 138 | 23.402 | < 0.0001 |
|  | Latitudinal origin | 1, 138 | 350.039 | < 0.0001 |
|  | Microbiome:Salinity | 2, 138 | 4.273 | 0.0158 |
|  | Microbiome x Latitudinal origin | 1, 138 | 25.326 | < 0.0001 |
|  | Salinity x Latitudinal origin | 2, 138 | 9.046 | 0.0002 |
|  | Microbiome x Salinity x Latitudinal origin | 2, 138 | 3.357 | 0.0377 |
| Plant final survival | (Intercept) | 1, 138 | 1543.664 | < 0.0001 |
|  | Microbiome | 1, 138 | 12.3635 | 0.0025 |
|  | Salinity | 2, 138 | 22.7405 | < 0.0001 |
|  | Latitudinal origin | 1, 138 | 3.8564 | 0.0652 |
|  | Microbiome:Salinity | 2, 138 | 2.1268 | 0.1482 |
|  | Microbiome x Latitudinal origin | 1, 138 | 4.5101 | 0.0478 |
|  | Salinity x Latitudinal origin | 2, 138 | 0.1436 | 0.8672 |
|  | Microbiome x Salinity x Latitudinal origin | 2, 138 | 4.4823 | 0.0263 |

SUPPLEMENTARY TABLE 2 | Analysis of variance of the LMM models that were fitted independently for the experimental plants within each level of saline stress. In this case, each analysis indicates the influence of the symbiotic condition on the observed response within a given level of saline stress (Control, 200 mM or 400 mM NaCl), and its variation among cultivars from distinct latitudinal origins (19°, 29°, 34°, 35°, and 39° S). The significance of the interaction term suggests that the latitudinal pattern of cultivar responses depends on the symbiotic status of the plant (E^+^ or E^-^). *d.f*.: degrees of freedom, *F*: F-statistic, *p*: probability value. A p-value less than 0.05 indicates that the observed effect of a factor or its interaction is statistically significant.

| Response | Factor | *d.f* | *F* - value | *p*-value |
| --- | --- | --- | --- | --- |
| F_v_/F_m_ Control | (Intercept) | 1, 106 | 651584.9 | <.0001 |
|  | Microbiome | 1, 106 | 12.2 | 0.0007 |
|  | Latitudinal origin | 1, 106 | 8.5 | 0.0044 |
|  | Microbiome x Latitudinal origin | 1, 106 | 8 | 0.0056 |
| F_v_/F_m_  200 mM NaCl | (Intercept) | 1, 116 | 15932.859 | <.0001 |
|  | Microbiome | 1, 116 | 29.507 | <.0001 |
|  | Latitudinal origin | 1, 116 | 154.332 | <.0001 |
|  | Microbiome x Latitudinal origin | 1, 116 | 18.083 | <.0001 |
| F_v_/F_m_  400 mM NaCl | (Intercept) | 1, 116 | 6319.767 | <.0001 |
|  | Microbiome | 1, 116 | 26.549 | <.0001 |
|  | Latitudinal origin | 1, 116 | 7.289 | 0.008 |
|  | Microbiome x Latitudinal origin | 1, 116 | 6.884 | 0.0099 |
| *NHX1* Control | (Intercept) | 1, 46 | 7118.208 | <.0001 |
|  | Microbiome | 1, 46 | 38.93 | <.0001 |
|  | Latitudinal origin | 1, 46 | 0.001 | 0.9812 |
|  | Microbiome x Latitudinal origin | 1, 46 | 2.398 | 0.1283 |
| *NHX1*  200 mM NaCl | (Intercept) | 1, 46 | 2516.7963 | <.0001 |
|  | Microbiome | 1, 46 | 6.3924 | 0.015 |
|  | Latitudinal origin | 1, 46 | 27.2256 | <.0001 |
|  | Microbiome x Latitudinal origin | 1, 46 | 0.9696 | 0.3299 |
| *NHX1*  400 mM NaCl | (Intercept) | 1, 46 | 1604.7575 | <.0001 |
|  | Microbiome | 1, 46 | 4.0638 | 0.0497 |
|  | Latitudinal origin | 1, 46 | 14.7712 | 0.0004 |
|  | Microbiome x Latitudinal origin | 1, 46 | 0.3333 | 0.5666 |
| Seed weight Control | (Intercept) | 1, 46 | 5502.434 | <.0001 |
|  | Microbiome | 1, 46 | 0.139 | 0.711 |
|  | Latitudinal origin | 1, 46 | 65.30 | <.0001 |
|  | Microbiome x Latitudinal origin | 1, 46 | 6.518 | 0.0141 |
| Seed weight 200 mM NaCl | (Intercept) | 1, 46 | 4931.22 | <.0001 |
|  | Microbiome | 1, 46 | 47.43 | <.0001 |
|  | Latitudinal origin | 1, 46 | 12.89 | 0.0008 |
|  | Microbiome x Latitudinal origin | 1, 46 | 10.84 | 0.0019 |
| Seed weight 400 mM NaCl | (Intercept) | 1, 46 | 4543.03 | <.0001 |
|  | Microbiome | 1, 46 | 77.064 | <.0001 |
|  | Latitudinal origin | 1, 46 | 17.666 | <.0001 |
|  | Microbiome x Latitudinal origin | 1, 46 | 24.557 | <.0001 |
| Seed protein Control | (Intercept) | 1, 46 | 2210.7327 | <.0001 |
|  | Microbiome | 1, 46 | 4.3821 | 0.0419 |
|  | Latitudinal origin | 1, 46 | 119.6595 | <.0001 |
|  | Microbiome x Latitudinal origin | 1, 46 | 0.3909 | 0.5349 |
| Seed protein 200 mM NaCl | (Intercept) | 1, 46 | 4238.494 | <.0001 |
|  | Microbiome | 1, 46 | 53.321 | <.0001 |
|  | Latitudinal origin | 1, 46 | 116.518 | <.0001 |
|  | Microbiome x Latitudinal origin | 1, 46 | 26.788 | <.0001 |
| Seed protein, 400 mM NaCl | (Intercept) | 1, 46 | 4392.693 | <.0001 |
|  | Microbiome | 1, 46 | 68.459 | <.0001 |
|  | Latitudinal origin | 1, 46 | 137.495 | <.0001 |
|  | Microbiome x Latitudinal origin | 1, 46 | 21.329 | <.0001 |
| Plant final survival Control | (Intercept) | 1, 6 | 2366.3956 | <.0001 |
|  | Microbiome | 1, 6 | 3.5006 | 0.1105 |
|  | Latitudinal origin | 1, 6 | 6.6007 | 0.0424 |
|  | Microbiome x Latitudinal origin | 1, 6 | 2.2767 | 0.1821 |
| Plant final survival, 200 mM NaCl | (Intercept) | 1, 6 | 730.1053 | <.0001 |
|  | Microbiome | 1, 6 | 2.5263 | 0.1631 |
|  | Latitudinal origin | 1, 6 | 2.1849 | 0.1898 |
|  | Microbiome x Latitudinal origin | 1, 6 | 1.9203 | 0.2151 |
| Plant final survival, 400 mM NaCl | (Intercept) | 1, 6 | 163.27301 | <.0001 |
|  | Microbiome | 1, 6 | 6.92074 | 0.039 |
|  | Latitudinal origin | 1, 6 | 0.25794 | 0.6297 |
|  | Microbiome x Latitudinal origin | 1, 6 | 5.74743 | 0.0535 |

SUPPLEMENTARY TABLE 3 **|** Estimated slopes of the fitted regressions derived from each of the five general LMM models evaluated in this study (Table 1), along with their statistical significance (i.e., testing whether slopes differ, or not, from zero). ***d.f*.**: degrees of freedom, **CI**: confidence intervals (95 %), ***t***: *t* statistic, ***p***: probability value. A p-value less than 0.05 indicates that the observed effect of a factor or its interaction is statistically significant.

| Response | Salinity treatment | Symbiotic status | Estimated slope | *d.f.* | Lower CI | Upper CI | *t*  ratio | *p*  value |
| --- | --- | --- | --- | --- | --- | --- | --- | --- |
| F_v_/F_m_ | 200 mM NaCl | E- | -5.54E-03 | 338 | -0.0075 | -0.0035 | -5.554 | < 0.0001 |
|  | 200 mM NaCl | E+ | -1.13E-02 | 338 | -0.0132 | -0.0093 | -11.33 | < 0.0001 |
|  | 400 mM NaCl | E- | -7.51E-05 | 338 | -0.0020 | 0.0018 | -0.075 | 0.94 |
|  | 400 mM NaCl | E+ | -5.26E-03 | 338 | -0.0072 | -0.0032 | -5.269 | < 0.0001 |
|  | Control | E- | 7.72E-04 | 338 | -0.0013 | 0.0028 | 0.741 | 0.4589 |
|  | Control | E+ | 1.11E-05 | 338 | -0.0020 | 0.0021 | 0.011 | 0.9915 |
| *NHX1* | 200 mM NaCl | E- | -0.0198 | 138 | -0.0330 | -0.0065 | -2.957 | 0.0037 |
|  | 200 mM NaCl | E+ | -0.0289 | 138 | -0.0422 | -0.0157 | -4.333 | < 0.0001 |
|  | 400 mM NaCl | E- | -0.0209 | 138 | -0.0342 | -0.0077 | -3.137 | 0.0021 |
|  | 400 mM NaCl | E+ | -0.0284 | 138 | -0.0416 | -0.0152 | -4.246 | < 0.0001 |
|  | Control | E- | 0.0030 | 138 | -0.0102 | 0.0163 | 0.456 | 0.649 |
|  | Control | E+ | -0.0031 | 138 | -0.0163 | 0.0101 | -0.47 | 0.6388 |
| Seed weight | 200 mM NaCl | E- | 0.0013 | 138 | -0.0108 | 0.0133 | 0.211 | 0.8334 |
|  | 200 mM NaCl | E+ | 0.0296 | 138 | 0.0176 | 0.0417 | 4.864 | < 0.0001 |
|  | 400 mM NaCl | E- | -0.0029 | 138 | -0.015 | 0.0091 | -0.482 | 0.6302 |
|  | 400 mM NaCl | E+ | 0.0358 | 138 | 0.0237 | 0.0478 | 5.873 | < 0.0001 |
|  | Control | E- | 0.0259 | 138 | 0.0138 | 0.0379 | 4.245 | < 0.0001 |
|  | Control | E+ | 0.0497 | 138 | 0.0377 | 0.0618 | 8.166 | < 0.0001 |
| Seed protein | 200 mM NaCl | E- | 0.1380 | 138 | 0.0540 | 0.2220 | 3.249 | 0.0015 |
|  | 200 mM NaCl | E+ | 0.3920 | 138 | 0.3082 | 0.4760 | 9.236 | < 0.0001 |
|  | 400 mM NaCl | E- | 0.1700 | 138 | 0.0855 | 0.2530 | 3.992 | 0.0001 |
|  | 400 mM NaCl | E+ | 0.3900 | 138 | 0.3058 | 0.4740 | 9.18 | < 0.0001 |
|  | Control | E- | 0.4040 | 138 | 0.3198 | 0.4880 | 9.509 | < 0.0001 |
|  | Control | E+ | 0.4530 | 138 | 0.3688 | 0.5370 | 10.66 | < 0.0001 |
| Plant final survival | 200 mM NaCl | E- | -0.0352 | 18 | -1.394 | 1.3240 | -0.054 | 0.9572 |
|  | 200 mM NaCl | E+ | -1.0909 | 18 | -2.45 | 0.2680 | -1.687 | 0.1089 |
|  | 400 mM NaCl | E- | 1.2176 | 18 | -0.141 | 2.5760 | 1.883 | 0.0760 |
|  | 400 mM NaCl | E+ | -1.8722 | 18 | -3.231 | -0.5130 | -2.895 | 0.0097 |
|  | Control | E- | -1.0557 | 18 | -2.414 | 0.3030 | -1.632 | 0.1200 |
|  | Control | E+ | -0.2745 | 18 | -1.633 | 1.0840 | -0.424 | 0.6763 |

**SUPPLEMENTARY FIGURE 1 |** Heatmap depicting the estimated physiological plant activity performance calculated using the proposed PR model (Equation 1). Lower values indicate a lower estimated physiological plant activity for each genotype under different experimental conditions (endophyte status and NaCl concentration).


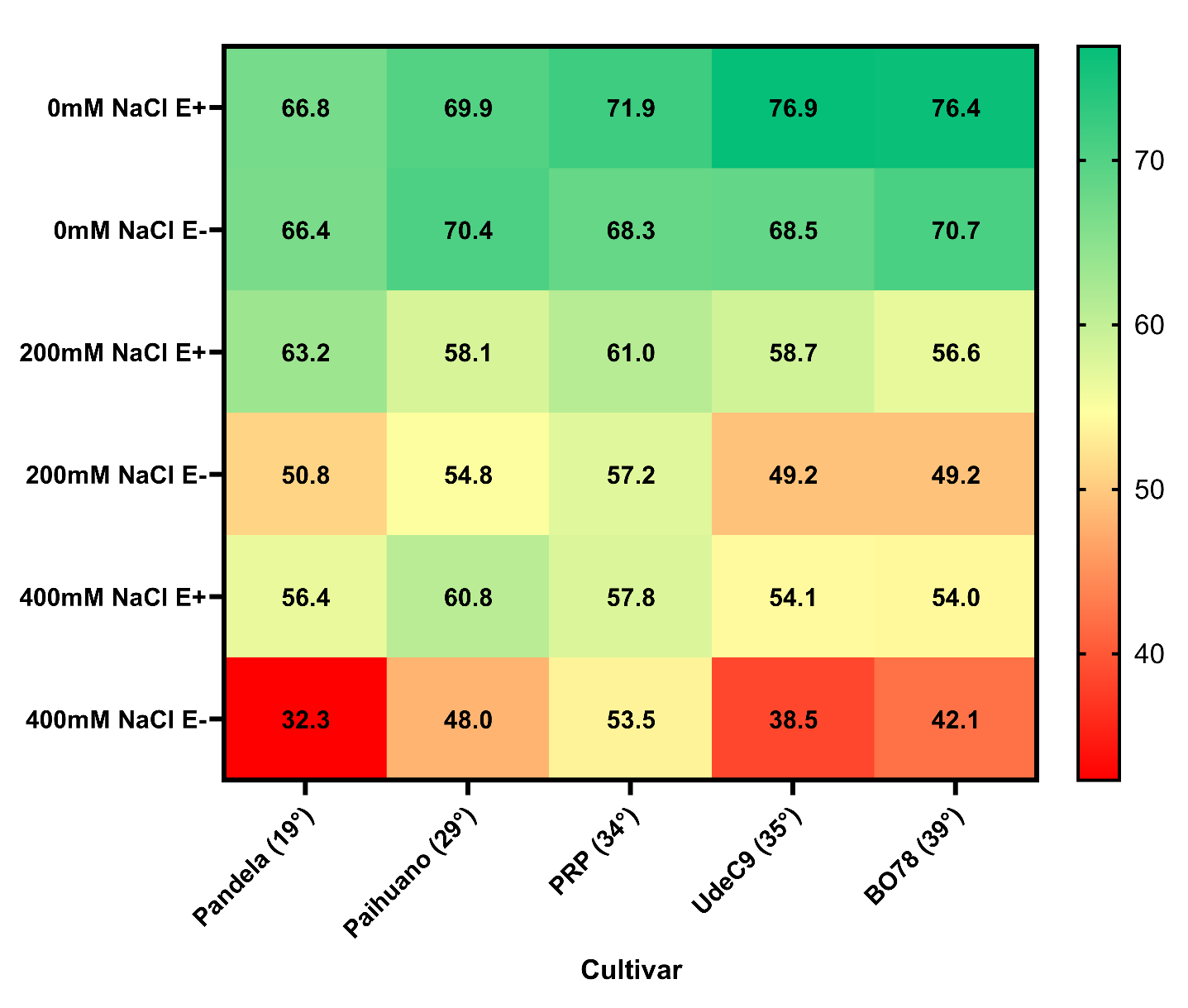

Supplement: Supplementary file 1 [file DataSheet1.docx]
